# A multivalent T-antigen-based vaccine for Group A Streptococcus

**DOI:** 10.1038/s41598-021-83673-4

**Published:** 2021-02-23

**Authors:** Jacelyn M. S. Loh, Tania Rivera-Hernandez, Reuben McGregor, Adrina Hema J. Khemlani, Mei Lin Tay, Amanda J. Cork, Jeremy M. Raynes, Nicole J. Moreland, Mark J. Walker, Thomas Proft

**Affiliations:** 1 Department of Molecular Medicine & Pathology, School of Medical Sciences, The University of Auckland, Private Bag 92019, Auckland, New Zealand; 2 Maurice Wilkins Centre for Molecular Biodiscovery, Auckland, New Zealand; 3 Australian Infectious Diseases Research Centre and School of Chemistry and Molecular Biosciences, The University of Queensland, St Lucia, QLD Australia; 4 Present Address: Cátedras CONACYT—Unidad de Investigación Médica en Inmunoquímica, Hospital de Especialidades del Centro Médico Nacional Siglo XXI, Instituto Mexicano del Seguro Social, Mexico City, Mexico; 5 Present Address: Monash University, Clayton Campus, Melbourne, VIC Australia

**Keywords:** Bacterial infection, Protein vaccines, Bacteria

## Abstract

Pili of Group A Streptococcus (GAS) are surface-exposed structures involved in adhesion and colonisation of the host during infection. The major protein component of the GAS pilus is the T-antigen, which multimerises to form the pilus shaft. There are currently no licenced vaccines against GAS infections and the T-antigen represents an attractive target for vaccination. We have generated a multivalent vaccine called TeeVax1, a recombinant protein that consists of a fusion of six T-antigen domains. Vaccination with TeeVax1 produces opsonophagocytic antibodies in rabbits and confers protective efficacy in mice against invasive disease. Two further recombinant proteins, TeeVax2 and TeeVax3 were constructed to cover 12 additional T-antigens. Combining TeeVax1–3 produced a robust antibody response in rabbits that was cross-reactive to a full panel of 21 T-antigens, expected to provide over 95% vaccine coverage. These results demonstrate the potential for a T-antigen-based vaccine to prevent GAS infections.

## Introduction

*Streptococcus pyogenes*, also known as Group A Streptococcus (GAS), is a human pathogen that is estimated to cause over 500,000 deaths annually^1^. Approximately one-third of these deaths are caused by severe invasive infections, while the majority are attributed to rheumatic heart disease (RHD). The severe damage to the heart seen in RHD patients is generally preceded by episodes of Acute Rheumatic Fever (ARF), an autoimmune disease triggered by multiple, untreated, superficial GAS infections such as pharyngitis or impetigo^2–4^. While ARF and RHD rates have been decreasing in most high-income settings, they continue to cause significant morbidity and mortality in low income regions of the world. Disproportionally high rates are also reported in Indigenous communities within countries such as New Zealand and Australia^5^. Large-scale sore-throat management programmes to control ARF in New Zealand have shown to be resource intensive and unlikely to be sustainable in low income settings^6^, and a vaccine is seen as a feasible and cost-effective solution for controlling disease long term^7,8^.

There are relatively few products in the GAS vaccine pipeline with only four, all based on the M-protein, having completed phase I clinical trials^9–12^. The M-protein is a major virulence determinant of GAS and evidence suggests that immune responses elicited to the M-protein can be protective^13,14^. However, with over 200 allelic variants of the *emm* gene (encodes the M-protein), achieving broad coverage in low income settings with high strain diversity remains a hurdle for M-type specific vaccines^15,16^. An approach based on the conserved C-repeat region of the M-protein negates the coverage issue, though questions remain around the immune accessibility of this region, especially in strains with a hyaluronic acid capsule^17^. Large, impure doses of a crude M-protein vaccine was associated with the development of ARF in historical trials^18,19^, which has further impeded vaccine development.

The pilus of GAS represents an attractive alternative target for vaccination. Pili are surface-exposed virulence factors that are involved in adhesion, colonisation, and immune evasion^20–23^. Targeting the pilus through vaccination using recombinant pilus proteins has been shown to be beneficial against type-specific infection in animal models^24,25^. However, the issue of antigenic variation of pilus proteins amongst different strains of GAS needs to be addressed. The main protein component of the pilus, the T-antigen, polymerises to form the elongated (> 1 µm long) pilus fibre (Fig. S1). The T-antigen is expressed as a precursor protein with an N-terminal secretion signal and a C-terminal sortase domain. An operon-encoded sortase recognises the sortase domain and covalently links up to ~ 100 monomeric T-antigens^25,26^. Most of the T-antigens consist of a two-domain protein structure, although some T-antigens are known or predicted to be three-domain (T6, T2, T23, T25) or four-domain (T4) proteins^26,27^. Historically, the T-antigen has been used as a supplementary serotyping tool to classify GAS strains. Though more recently, with the shift to routine genotypic *emm*-typing of clinical isolates in circulation, relatively few epidemiological studies have used T-serotyping. In many cases T-type (or *tee*-genotype) are highly linked^28^ and *tee*-type can be inferred from *emm*-type—for example all *emm*-1 strains carry the *tee*-1 gene. However, as *tee*-types are far less diverse than *emm*-types, the same *tee*-type can be found in multiple *emm*-type strains e.g. *tee-*11 is found in *emm-*11, 44, 78, and 89 strains. Based on inference from large-scale *emm*-typing surveillance studies in high-income countries, *tee*-1, 12, and 28 are the predominant *tee*-types in circulation, whereas in low-income countries a single-type dominance is not apparent (reviewed by Efstratiou et al.^29^). Previous sequence analysis of the *tee* gene from approximately 100 GAS isolates revealed 18 distinct *tee*-types and three sub-types, with little to no variation within each *tee*-type^30^. Inclusion of all 18 T-antigens into a vaccine is expected to provide greater than 95% coverage of GAS strains circulating globally.

Using a bioinformatic approach, we have designed a recombinant multivalent protein vaccine by fusing immunogenic domains of different T-antigen variants. The first of our three proposed fusion proteins, named TeeVax1, contains protein domains from six T-antigen variants (T28.1, T12, T1, T5, T18.1, T11). We demonstrate that immunisation with TeeVax1 can generate opsonophagocytic antibodies and provides protective efficacy in a mouse model of GAS invasive disease. A further two multivalent recombinant proteins (TeeVax2 and TeeVax3) were constructed by similar means, covering the remaining major T-antigen clades (Fig. S2). We show that immunising with a mixture of all three TeeVax proteins can generate broadly cross-reactive antibodies against the full panel of 21 known T-antigens. Importantly, all three TeeVax proteins are soluble and can be produced in *E. coli* with high yield, making them suitable for large scale vaccine production.

## Results

### Cloning and expression of TeeVax1

**Figure 1:**
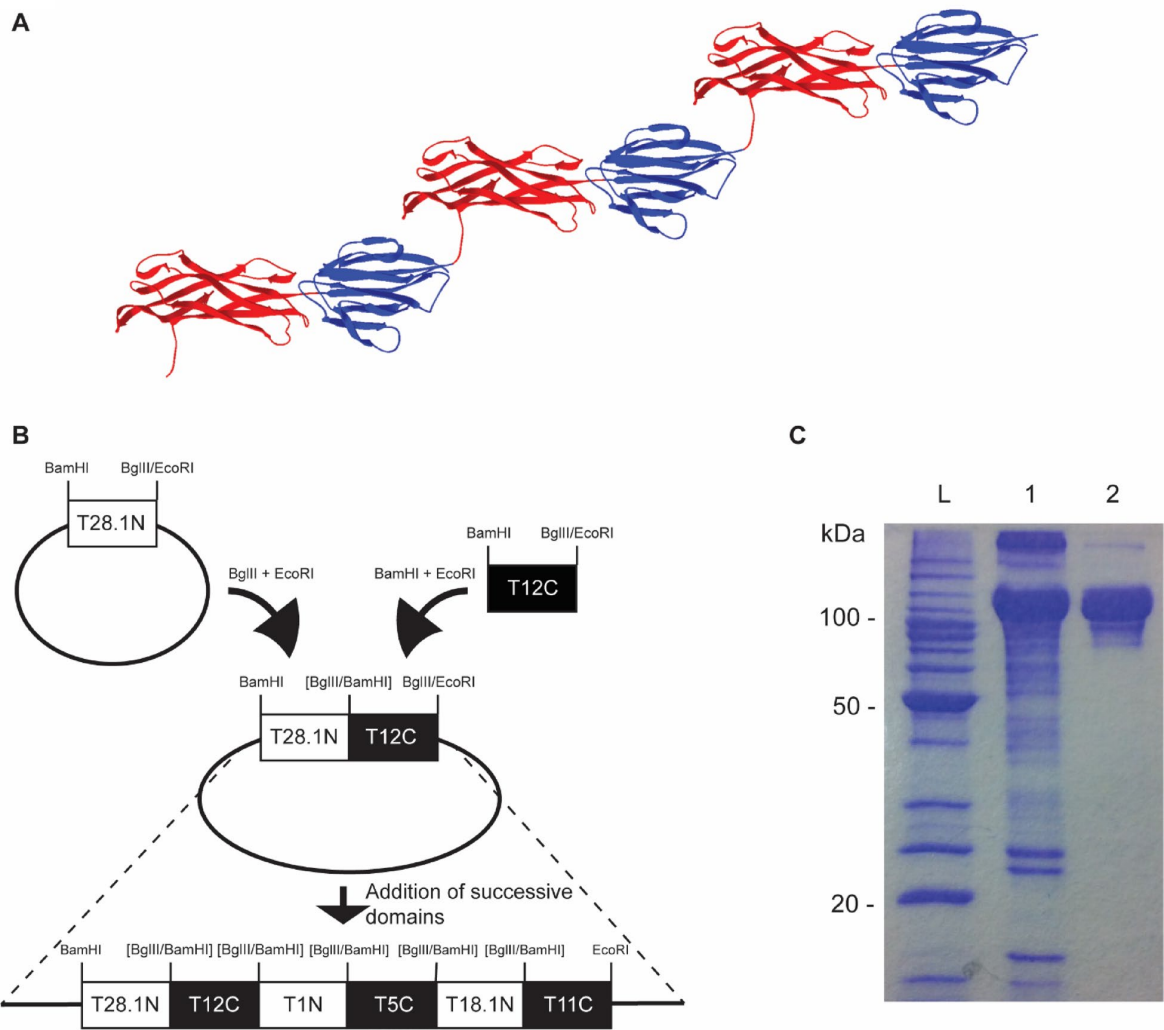
Construction of TeeVax1. (**A**) Structural model of TeeVax1 showing predicted N-terminal (red) and C-terminal (blue) domain fusions of six T-antigens. (**B**) Cloning strategy used to join successive T-antigen domains from different strains. Restriction sites in brackets denote irreversible ligation using compatible sticky ends. (**C**) 12.5% SDS-PAGE analysis of TeeVax1 stained with Coomassie blue dye. L = Benchmark protein ladder, 1 = After nickel-affinity chromatography, 2 = After size-exclusion chromatography.

Previous bioinformatic analysis of two-domain T-antigens revealed a highly conserved core decorated by surface variation along the full length of the protein, with no apparent dominant region^31^. We therefore designed a multivalent vaccine that included whole T-antigen domains to maximise both the inclusion of protective epitopes and protein yield. TeeVax1 was constructed as a fusion of alternating N- and C-terminal domains from six different two-domain T-antigens to resemble a hybrid pilus fibre. Primers were designed based on the predicted boundaries for each domain generated from these models^31^. The BamHI restriction site was engineered at the 5′ end, and BglII and EcoRI restriction sites were engineered at the 3′ end of each domain, introduced by the PCR primers (Table S2). BamHI and BglII have compatible cohesive ends which cannot be re-cleaved after ligation. This allowed successive sequential addition of each *tee* domain into the vector by performing appropriate BamHI/EcoRI and BglII/EcoRI digests (Fig. 1). TeeVax1 comprised of T28.1N, T12C, T1N, T5C, T18.1N, and T11C, cloned sequentially and expressed in *E. coli* BL21(DE3) pLysS producing a protein with a predicted molecular weight of 102 kDa. Purification by immobilised metal affinity and size exclusion chromatography resulted in a final yield of approximately 10 mg/L bacterial culture (Fig. 1C).

### Antibody responses following TeeVax1 immunisation of rabbits

**Figure 2:**
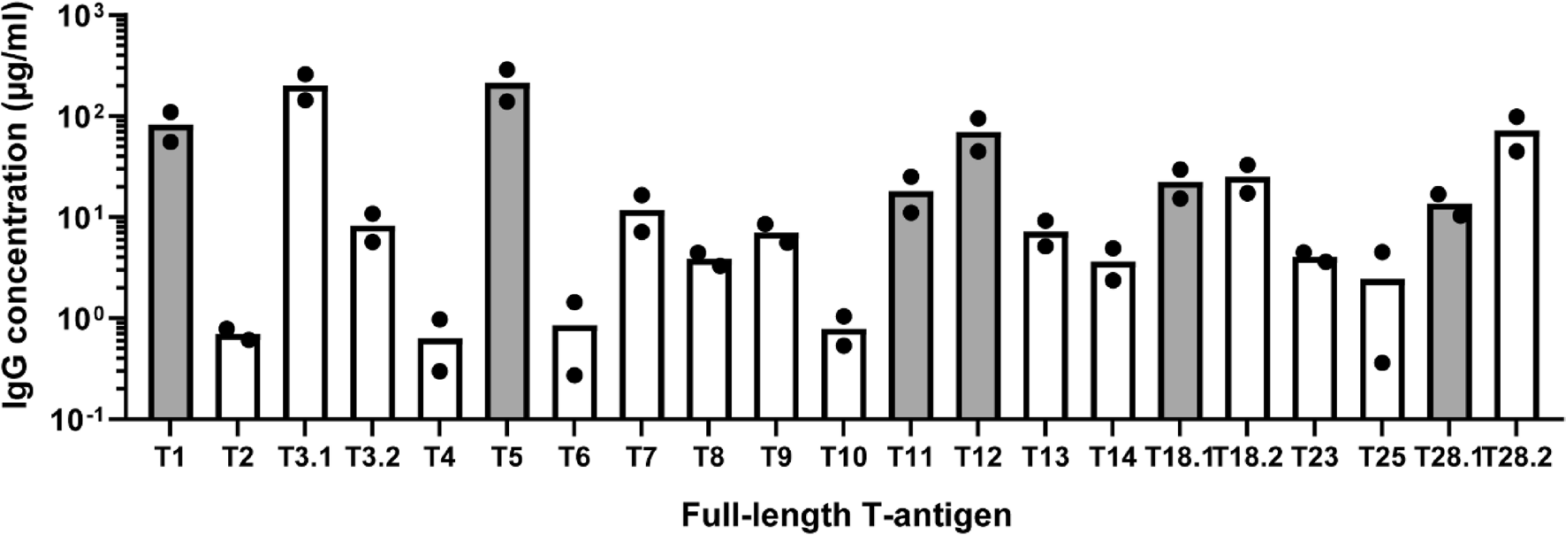
Serum antibody responses to TeeVax1 in rabbits. Sera from rabbits (n = 2) immunised with TeeVax1 were analysed by ELISA. IgG concentration to each specific T-antigen was interpolated using a known standard. Each biological replicate is plotted (circles), with bar representing the mean IgG concentration. Grey bars represent T-antigens present in the TeeVax1 construct and white bars represent T-antigens absent in TeeVax1.

Purified TeeVax1 was used to immunise rabbits. Antisera collected 2 weeks after the final boost were tested by ELISA against a panel of 21 full-length recombinant T-antigens (Fig. 2). Specific serum IgG responses towards cognate T-antigens (T28.1, T12, T1, T5, T18.1, T11) were in the range of 10–280 µg/ml. The lowest response was seen towards T11, and the highest to T5. Extensive cross-reactivity was seen towards T3.1, with IgG concentrations averaging 200 µg/ml. Cross-reactivity to closely related T-antigens, T28.2 and T18.2, was also observed. Cross-reactivity to all other T-antigens were low or not detected.

### Opsonophagocytic killing assay (OPKA) of TeeVax1 antiserum

**Figure 3:**
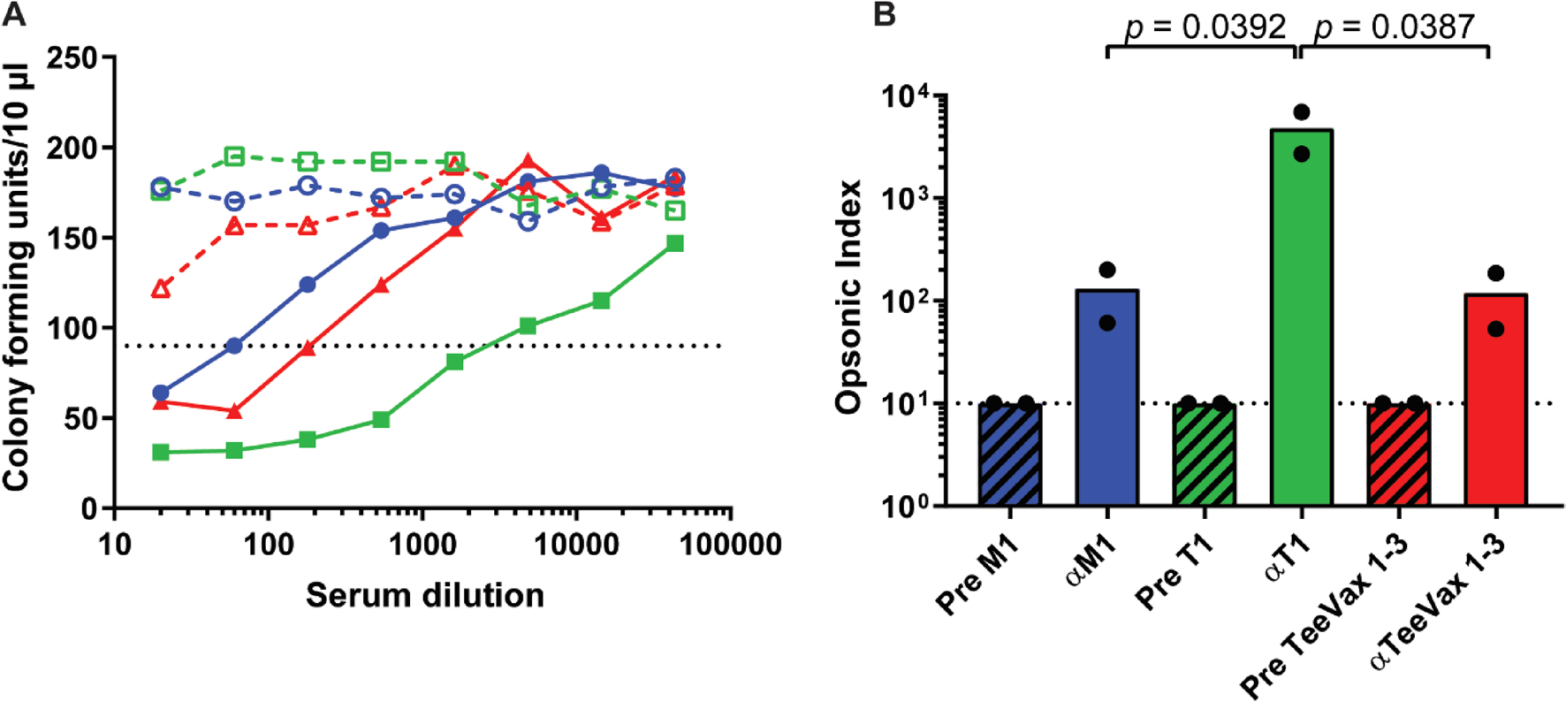
OPKA of rabbit antiserum. Sera from immunised rabbits were tested in an OPKA against GAS SF370 (M1T1 strain). (**A**) Representative line graph showing CFU recovered after incubation with serial dilutions of M1 (blue), T1 (green), and TeeVax1 (red) antiserum. Open symbols indicate pre-immune sera, solid symbols indicate immune sera. Serum dilution resulting in 50% killing is denoted by the point at which the curve crosses the dotted line which is used to calculate the opsonic index. (**B**) Opsonic index (n = 2) calculated from the serum dilution resulting in 50% killing. Each biological replicate is plotted (circles), with bar representing the mean. Hashed bars indicate pre-immune serum (Pre), solid bars indicate immune serum. Dashed line denotes the limit of detection.

Rabbit antiserum was tested for opsonophagocytic function in a standardised OPKA against GAS SF370 (a M1T1 strain). Antiserum from a rabbit immunised with M1-protein was used as a positive control and pre-immune serum as a negative control. Opsonophagocytic killing was significantly higher with anti-T1 serum compared with anti-M1 and anti-TeeVax1, with opsonophagocytic indices of 4802, 131 and 119, respectively (Fig. 3).

### Vaccine efficacy in humanised plasminogen transgenic mice

**Figure 4:**
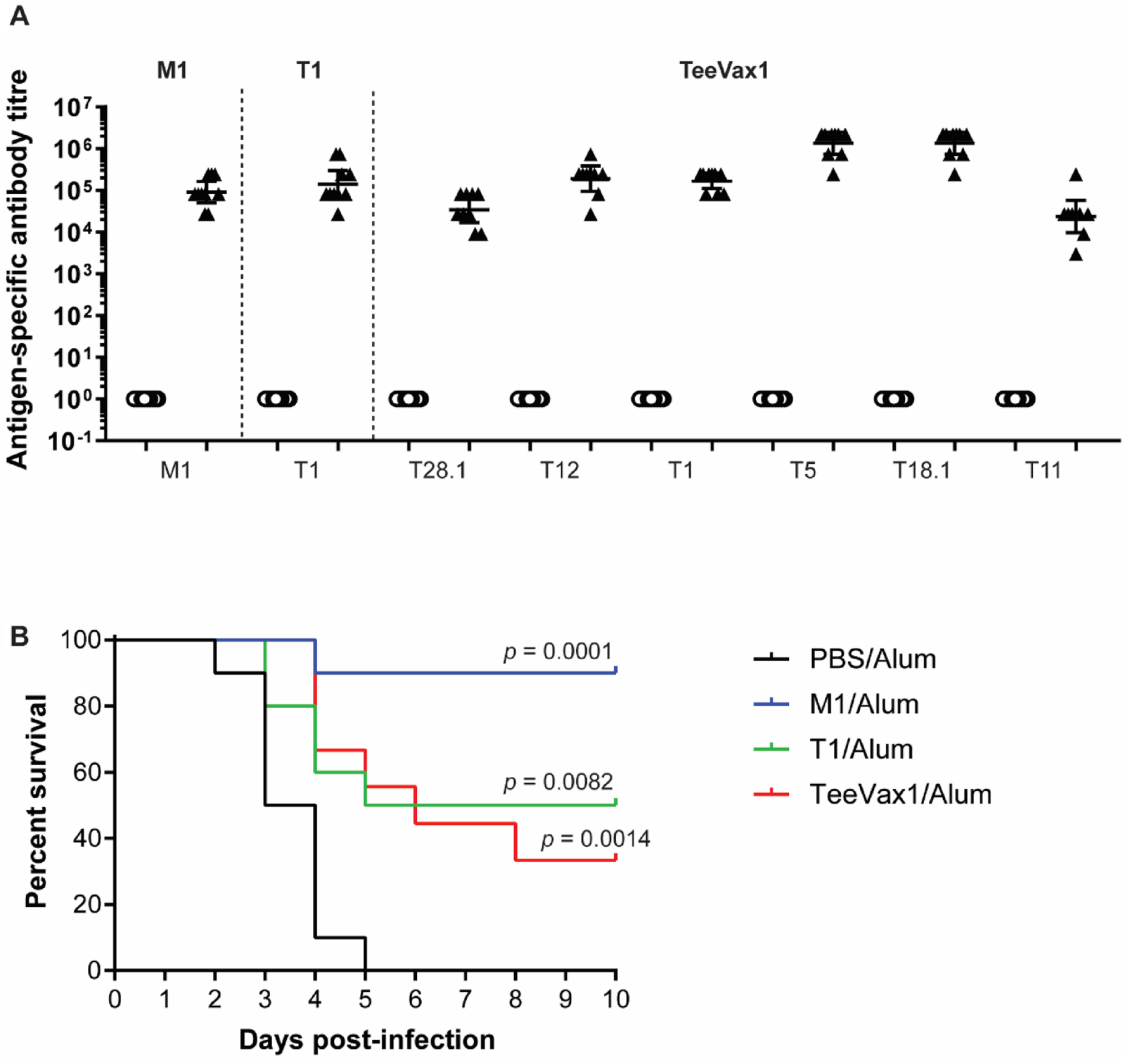
Mouse challenge model. *Alb*PLG1 mice were immunised i.m. with M1/alum (n = 10), T1/alum (n = 10), TeeVax1/alum (n = 9) or PBS/alum (n = 10). Two weeks after the final immunisation, all mice were challenged s.c. with GAS 5448 and monitored for 10 days. (**A**) Antibody titres from day 35 sera of immunised mice prior to challenge were measured by ELISA against cognate antigens. Circles = titre from PBS/alum-immunised mice, triangles = respective antigen-immunised mice. (**B**) Kaplan–Meier survival analysis of immunised mice challenged with GAS 5448. All groups were significantly protected from challenge compared to PBS control (log-rank Mantel-Cox test).

Vaccine efficacy was assessed using the humanised plasminogen transgenic mouse model of invasive disease^32^. Mice were immunised with TeeVax1, T1-antigen, M1-protein, or PBS (sham) and challenged subcutaneously with GAS strain 5448 (a M1T1 invasive disease isolate optimised for this model). Prior to challenge, specific serum antibody responses were measured by ELISA. All vaccinations produced significantly higher IgG titres towards their cognate antigen(s) compared with PBS-immunised control mice (Fig. 4A). TeeVax1-immunised mice showed strong antibody responses to all six T-antigens contained in the fusion protein. There was no significant difference between anti-T1 titres from mice immunised with full-length T1 versus TeeVax1. Following challenge with GAS, survival was monitored for 10 days. All vaccine antigens provided significant protection against challenge (p < 0.01) compared to PBS-immunised mice (Fig. 4B). Full-length M1-protein was able to confer near complete protection with 90% survival as observed previously^33,34^. Both TeeVax1 and T1 provided moderate protection with 33% and 50% survival after 10 days, respectively.

### Construction of TeeVax2 and TeeVax3

Evidence of protection with TeeVax1 warranted the construction of two additional TeeVax proteins, TeeVax2 and TeeVax3, to extend vaccine strain coverage. TeeVax2 was designed and constructed in a similar manner to TeeVax1, with alternating N- or C-terminal domains from predicted two-domain T-antigens (T9, T3.2, T8, T10, T14, T7)*,* and terminating with the full-length T13 (both N- and C-terminal domains). T13 was included in its full-length form as terminating TeeVax2 with T13N or T13C resulted in a marked reduction in the expression of the fusion protein in *E. coli* (data not shown). The yield of TeeVax2 was approximately threefold higher than TeeVax1 at 30 mg/L of bacterial culture. TeeVax3 contained domains from the genetically divergent three-domain (T6, T2, T23, T25) and four-domain (T4) T-antigens. The crystal structure of T6 from GAS strain MGAS10394 (PDB ID 4P0D)^27^ was used to model and predict domain boundaries of T2, T23, and T25. T4 was modelled onto RrgB (PDB ID 3RPK)^35^, a four-domain pilin that forms the pilus backbone in *S. pneumoniae*^35^. The middle domain from T6, T2, T23, and T25, and the N-terminal domain from T4 were cloned successively into pPROEX-HTb. Previous structural analysis of T-antigens suggested that antigenic epitopes are found across entire surface of the T-antigen structure without any apparent dominant domain^27,31^. However, the N-terminal domain was chosen for T4, as previous studies on RrgB indicated that the N-terminal domain was as protective as the full-length protein^35^. As the joining of successive middle-domains did not closely resemble the native structure of the three- or four-domain T antigens, a short linker sequence consisting of five amino acids (GSGSG) was introduced between each domain encoded in the PCR primers used during amplification. This was anticipated to provide some separation between the individual domains and promote protein folding. The yield of TeeVax3 was approximately twofold higher than TeeVax1 at 20 mg/L of bacterial culture.

### Antibody responses to TeeVax2, TeeVax3, and TeeVax1–3

**Figure 5:**
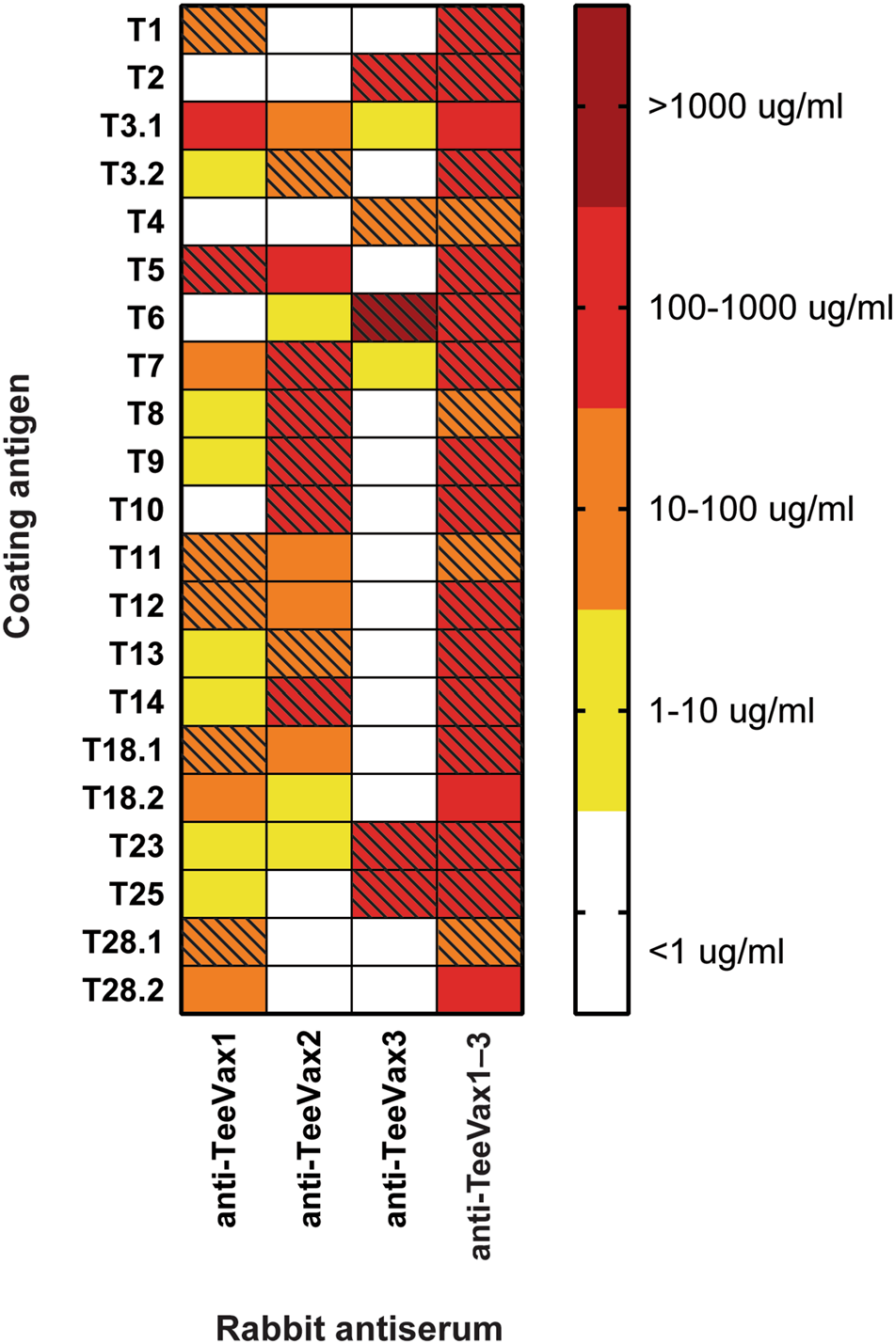
Antigen-specific IgG response of TeeVax proteins. Sera from rabbits immunised with TeeVax1 (n = 2), TeeVax2 (n = 1), TeeVax3 (n = 1) or an equimolar concentration of all three (TeeVax1–3) (n = 1) were analysed by ELISA. IgG concentration to each specific full-length T-antigen was interpolated using a known standard and presented as a heat-map. Hashing denotes homologous vaccine antigens.

Purified TeeVax proteins were used individually or in combination to immunise rabbits. Antisera collected 2 weeks after the final boost were measured by ELISA against a panel of 21 full-length recombinant T-antigens (Fig. 5). Antiserum from TeeVax2 produced specific antibodies to T-antigens contained within TeeVax2, as well as substantial cross-reactive antibodies to other two-domain T-antigens including T3.1, T11, T12, T18, and T28. TeeVax3 antiserum showed little or no cross-reactivity against the two-domain T-antigens but was reactive towards the T-antigens contained within TeeVax3. Importantly, antiserum from rabbits immunised with the mixture of all three TeeVax proteins combined (TeeVax1–3) reacted to the full panel of 21 T-antigens, including the three sub-types (T3.1, T18.2, T28.2) not included in any of the individual vaccines. Specific serum IgG concentrations ranged from 10 to 1200 µg/ml to individual T-antigens. Pre-immune serum contained < 1 µg/ml of T-antigen-specific IgG (data not shown). TeeVax1-3 antisera was also shown to be able to detect native pilus on the surface of GAS SF370 by flow cytometry (Fig. S3).

## Discussion

GAS is one of the top ten causes of death by an infectious agent worldwide and the need for a GAS vaccine is clear. Recent resurgence of serious invasive infections^36^ and the reports of frequent treatment failures despite GAS remaining sensitive to penicillin^37–39^ has added to renewed attention to achieving this goal. There are several reasons why vaccine development for GAS has been impeded thus far, including an incomplete understanding of how GAS triggers immunopathological conditions such as ARF^40^. It is unclear for example the extent to which certain virulence factors may elicit the production of cross-reactive antibodies to human proteins. The use of whole microorganisms is therefore impractical, turning the spotlight to well-defined subunit vaccines.

Here, we describe a type-specific vaccine based on the surface exposed pilus of GAS. We have previously shown that targeting the pilus can generate protective immune responses in a mouse colonisation model^24^. However, antigenic variation, while far more limited compared to the M-protein, needed to be addressed. The multivalent approach described here broadly targets the major backbone protein (the T-antigen) of the GAS pilus from multiple strains. Whole protein domains (~ 140 amino acids each) were genetically fused to form a recombinant, soluble chimera of the GAS pilus fibre, called TeeVax. TeeVax1, a protein covering six T-antigen serotypes, produced antibody responses against homologous antigens as well as cross-reactive antibodies to an additional three heterologous T-antigens. The response to T5 was by far the highest with over 200 µg/ml of specific IgG produced in rabbits. Similarly, high antibody levels were observed towards the T3.1 antigen, which is unsurprising given that it shares an identical C-terminal domain to T5^30^. The lowest response was to T11, which is likely due to the position of this domain at the carboxy-terminus of the fusion protein. A comparatively similar reduction in the antibody response to carboxy-terminal positioned T4 domain in TeeVax3 was also observed, possibly suggesting an enhanced cleavage/degradation by host proteases following injection. This observation is similar to those seen in the multivalent M-protein vaccines and may potentially be solved by re-iteration of the same domain at the N-terminus or addition of a protective “cap” protein at the C-terminus^41^. Response to the carboxy-terminal positioned T13 in TeeVax2 was not reduced, possibly because of shared cross-reactive epitopes with T3.2^31^; or because full-length T13 was incorporated in this chimera, rather than a single domain, and thus may serve as its own protective cap.

Opsonophagocytic function of rabbit antiserum was assessed against the M1T1 strain SF370 using the HL-60 OPKA^42^. TeeVax1 antiserum produced a similar opsonic index to M1-protein antiserum that was modest, but in the range reported in other studies using this assay^42,43^. T1 antiserum produced a far higher opsonic index which was expected due to the higher concentration of specific antibodies present in the T1-antiserum (~ 20-fold higher, data not shown). However we cannot exclude the possibility that antibodies to the C-terminal domain of T1 (that is not included in TeeVax1) are better opsonins. Whether TeeVax antisera is able to exert opsonophagocytic function on other GAS strains is the subject of future studies.

In mice, no difference between T1-specific antibody titres when immunising with T1 vs TeeVax1 was observed. This translated to a similar level of protection in the *Alb*PLG1 mouse challenge model of invasive disease. Immunisation with TeeVax1 or T1 significantly improved survival of mice in this model. Full-length M1-protein was superior for preventing invasive disease in this model, however, antibodies evoked by M-protein vaccination have been shown to cross-react with human proteins (reviewed by Guilherme et al.^44^), raising strong safety concerns. No such concerns have been reported for the T-antigen. In keeping, we conducted homology searches between the TeeVax proteins and human proteome using the Universal Protein Resource database (UniProt)^45^. Less than 35% identity was reported for sequence hits, and no human heart proteins were identified.

There is wide variation in reported protection of GAS vaccine candidates in murine models, with recent studies showing vaccines can be protective in one but not another model^33^. The same *Alb*PLG1 mouse invasive disease model used here to assess TeeVax, was previously used to assess other vaccine candidates. Of note, the J8 antigen adjuvanted with alum was not protective in this model^33^, nor was the alum-adjuvanted Combo#5 (containing five conserved GAS antigens: streptolysin O, *Streptococcus pyogenes* cell envelope proteinase, arginine deiminase, trigger factor, and Group A streptococcal C5a peptidase), or Group A carbohydrate vaccine candidates^33^. This highlights a potential protective benefit of TeeVax-alum over these vaccines against invasive disease. Nonetheless, protection using these vaccine candidates has been observed in other animal infection models^33,43,46,47^.

We have shown that TeeVax1–3 has broad coverage against a panel of 21 T-antigens, representative of > 95% of all GAS T-serotypes known to-date. Serum antibody concentrations to specific T-antigens were in many cases higher in the TeeVax1–3 combination compared to the corresponding single TeeVax protein, likely due to cross-reactive epitopes^31^. The GAS pilus can extend further from the cell surface than other GAS proteins, most likely penetrating the hyaluronic acid capsule, due to the multimeric nature of the T-antigen that forms the pilus fibre^48^. This makes it an attractive vaccine target due to the easy accessibility to the immune system. While we have yet to test TeeVax1–3 in an in vivo challenge model, this study clearly demonstrates the potential of creating a broadly protective vaccine against GAS by targeting pili.

## Methods

### Animal ethics

Procedures using humanised plasminogen *Alb*PLG1 mice were conducted according to the Australian Code for the Care and Use of Animals for Scientific Purposes at the University of Queensland, Australia, and approved by the University of Queensland Animal Ethics Committee (SCMB/136/16/NHMRC/BREED and SCMB/140/16/NHMRC). All other animal experiments were performed in the Vernon Jansen Unit at The University of Auckland, New Zealand, and were approved by The University of Auckland Animal Ethics Committee. This study was carried out in compliance with the ARRIVE guidelines.

### Bacteria and cell culture conditions

GAS strains used in this study are listed in Supplementary Table 1. GAS was cultured in Todd Hewitt broth supplemented with 0.5% yeast (THY) at 37 °C, under static conditions. *E. coli* DH5α and BL21(DE3) pLysS (Novagen) were cultured in LB at 37 °C at 200 rpm. Solid growth medium was prepared by adding 1.5% bacteriological agar. Growth medium was supplemented with 30 µg/ml chloramphenicol and 50 µg/ml ampicillin when required.

### Cloning and protein expression

The *tee* or *emm1* genes were amplified from genomic DNA between the predicted signal peptide cleavage site and sortase motif by PCR using iProof high-fidelity polymerase (Bio-rad) with primers listed in Supplementary Table 2. Genes were cloned into the expression vector pPROEX-HTb (ThermoFisher) or a modified pET-32a (Novagene) vector with a 3c cleavage site, and transformed into *E. coli* BL21(DE3) pLysS. For generation of the multivalent TeeVax constructs, domains were cloned successively using compatible overhangs generated by BamHI and BglII restriction digestion. Proteins were expressed by induction with 0.1 mM Isopropyl β-d-1-thiogalactopyranoside (IPTG) at 18 °C for 18 h. All proteins were purified following previously published protocols^27,31^. This included nickel-affinity chromatography using either a HisTrap HP column (GE Healthcare) on an AKTA FPLC or a gravity flow column with Ni^2+^-NTA sepharose 6 fast flow (GE healthcare) using buffers containing up to 250 mM imidazole in 20 mM sodium phosphate buffer pH 7.4/300 mM NaCl/1% glycerol. A second purification step was performed by size exclusion chromatography using a Superdex 200 increase 10/300 GL column (GE healthcare) in 20 mM sodium phosphate buffer pH 7.4/300 mM NaCl/1% glycerol.

### Rabbit immunisations

New Zealand white (NZW) rabbits were immunised subcutaneously on days 0, 14, and 28 with 100 µg of recombinant protein emulsified 1:1 with incomplete Freund’s adjuvant (IFA). Euthanasia was performed by pentobarbital injection. Antiserum was collected on day 42.

### Flow cytometry

GAS SF370 was grown to early exponential phase (OD_600nm_ = 0.2) then blocked with PBS/5 mM EDTA/3% fetal bovine serum (FBS). GAS was then resuspended in 1:10 rabbit serum diluted in wash buffer (PBS/5 mM EDTA/1% FBS) and incubated on ice for 30 min. Goat anti-rabbit IgG conjugated to Alexa Fluor 647 (Life Technologies) at 1:100 dilution was used for detection. A total of 10,000 events were acquired using a BD LSRII flow cytometer (BD Biosciences).

### Enzyme-linked immunosorbent assay (ELISA)

MaxiSorp plates (Nunc) were coated with 1 µg/ml full-length T-antigen in PBS overnight at 4 °C. Diluted antiserum was added to the coated wells and incubated for 3 h at room temperature. The wells were washed with phosphate-buffered saline/0.05% tween-20 (PBS-T), followed by incubation with goat anti-rabbit IgG-HRP (ThermoFisher) for 1 h. The wells were washed with PBS-T and incubated with 100 µl of 3,3,5,5-tetramethylbenzidine (ThermoFisher). The colour development was stopped with 100 µl of 1 M hydrochloric acid. Absorbance at 450 nm was determined using an EnSpire multilabel plate reader (Perkin Elmer). For rabbit antisera, antibody concentrations were interpolated from a standard curve generated using affinity-purified anti-T-antigen antibodies of known concentration. For mouse antisera (day 35), endpoint titres were determined as the minimum serum dilution above the control (absorbance of 1:100 dilution of pre-immune serum + 3 times the standard deviation).

### Opsonophagocytic killing assay (OPKA)

OPKAs were performed similarly to previously described^42,49^ using the M1T1 strain SF370. Human promyelocytic leukemia (HL-60) cells were differentiated by incubation in 0.8% dimethylformamide (DMF) at 37 °C with 5% CO_2_ for 3 or 4 days. HL-60 cells passed acceptance criteria for differentiated phenotype (> 55% CD45, < 12% CD71, < 10% dead cells) as assessed by flow cytometry and were then diluted in opsonisation buffer (5% v/v heat-inactivated FBS (HyClone), 0.1% w/v gelatin (Sigma) in Hanks’ balanced salt solution (HBSS) with Ca/Mg) to 1 × 10^7^ cells per ml. GAS SF370 was washed and diluted in opsonisation buffer to ~ 1.2 × 10^5^ CFU/ml and 10 µl of the bacterial suspension was incubated for 30 min at room temperature, at 700 rpm, with 20 µl of serially diluted heat-inactivated sera. Pre-diluted baby rabbit complement (10 µl) and differentiated HL-60 cells (40 µl) were added to each dilution of serum and incubated for 60 min at 37 °C with 5% CO_2_, at 700 rpm. Plates were then placed on ice for 20 min, and 10 µl from each well was spotted onto THY agar plates. An overlay agar (THY, 0.75% w/v bacteriological agar, 0.005% 2,3,5-tetraphenyltetrazolium chloride (Sigma)) was poured onto each plate and plates were incubated overnight at 37 °C with 5% CO_2_. The number of surviving colony forming units (CFU) was counted on a ProtoCOL3 automated colony counter (Synbiosis). The serum dilution resulting in 50% killing is used to calculate the opsonic index (OI). Opsotiter software was used to calculate the OI and level of non-specific killing (NSK)^49^. OPKAs met all acceptance criteria: non-specific killing < 35% and maximum killing of immune sera > 70%. Assays were conducted with two biological replicates using the same rabbit antisera samples.

### Invasive disease mouse model

Transgenic humanised plasminogen mice (n = 10) heterozygous for the human plasminogen gene (*Alb*PLG1) were immunised intramuscularly with 30 µg of protein adsorbed to alum (Alhydrogel [2%]; Brenntag) on days 0, 21 and 28. Serum samples were taken before each immunisation and on day 35. One mouse from the TeeVax1 immunised group was excluded from the challenge due to an unrelated rectal prolapse. On day 41 mice were challenged subcutaneously with 1.3 × 10^8^ CFU of GAS strain 5448 as previously described^50,51^, and survival was monitored for 10 days. Euthanasia was performed by CO_2_ asphyxiation.

### Statistical analysis

All statistical analysis was performed using GraphPad Prism software. Significance was determined using ordinary one-way ANOVA with Tukey’s multiple comparisons test, p < 0.05. Survival analysis was compared using the log-rank Mantel-Cox test.

## Supplementary Information

Supplementary Information.
