# Supplementary material for: A multivalent T-antigen-based vaccine for Group A Streptococcus
Source: Sci Rep. 2021 Feb 23;11:4353. doi: 10.1038/s41598-021-83673-4 (PMC7902606; doi:10.1038/s41598-021-83673-4)
Supplement: Supplementary file 1 — Supplementary Information. [file 41598_2021_83673_MOESM1_ESM.pdf]

Supplementary Data

A multivalent T-antigen-based vaccine for Group A Streptococcus

Jacelyn M. S. Loh, Tania Rivera-Hernandez, Reuben McGregor, Adrina Hema J. Khemlani, Mei Lin Tay, Amanda J. Cork, Jeremy Raynes, Nicole J. Moreland, Mark J. Walker, and Thomas Proft

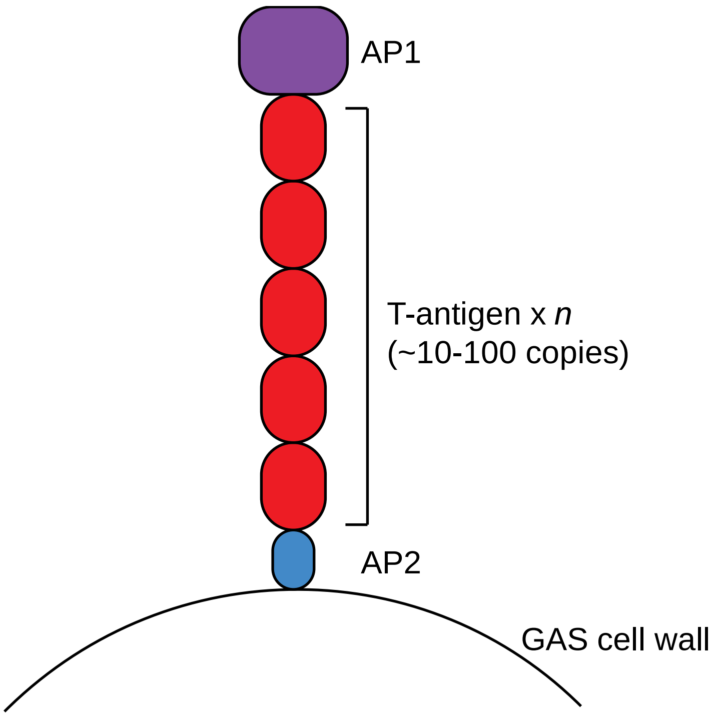

Fig. S1. Schematic diagram of the GAS pilus showing the T-antigen targetted for vaccination by TeeVax.
The GAS pilus is formed by covalent attachment of multiple pilus subunits to the cell wall. Strains express either one or two adaptor proteins (AP1 and AP2). Polymerisation of multiple copies of the T-antigen forms the pilus shaft.

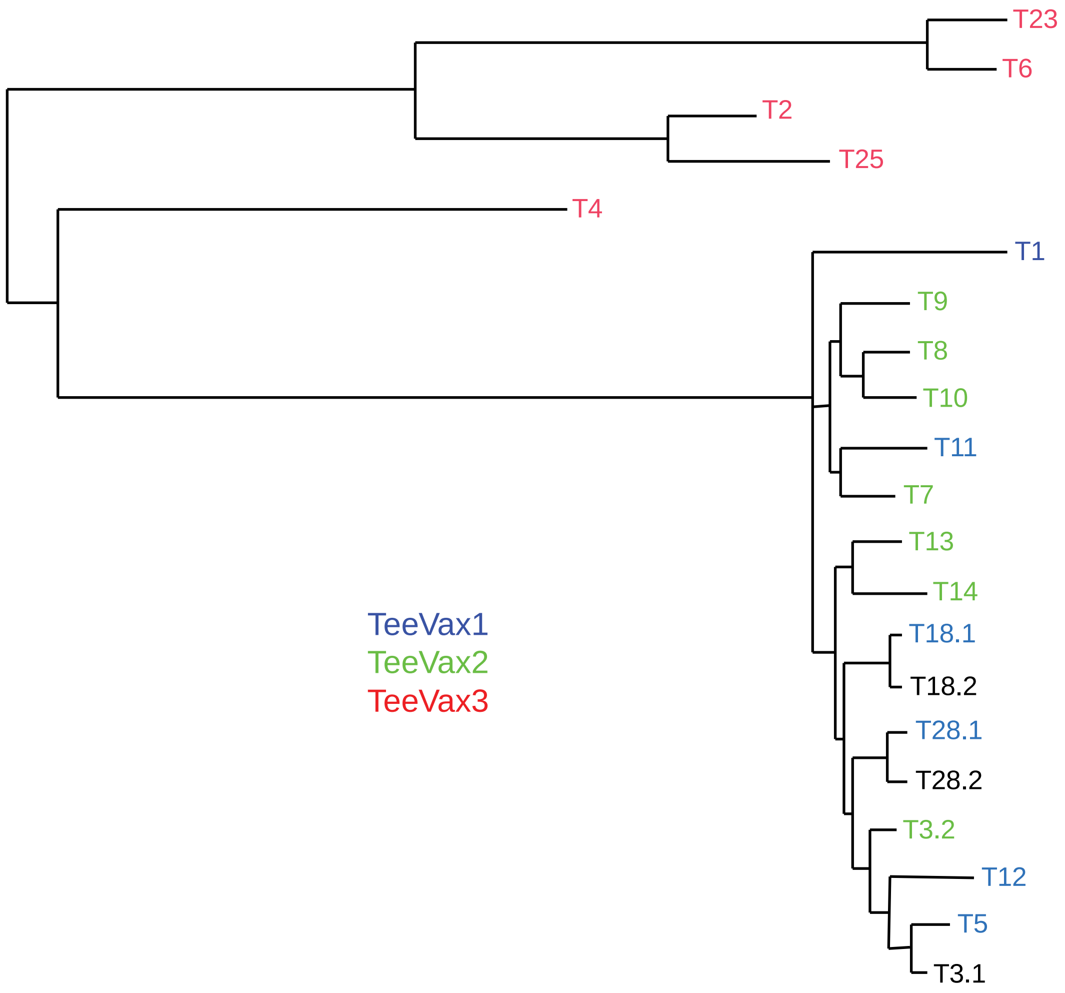

Fig. S2. Phylogenetic tree of T-antigen sequences.
The phylogenic tree was generated by multiple sequence alignment of T-antigen amino acid sequences from strains listed in Table S1 using ClustalW. The tree was inferred using PhyML v20160115 at [https://www.genome.jp/tools-bin/clustalw]. T-antigens incorporated into TeeVax1 (blue), TeeVax2 (green), and TeeVax3 (red) are indicated.

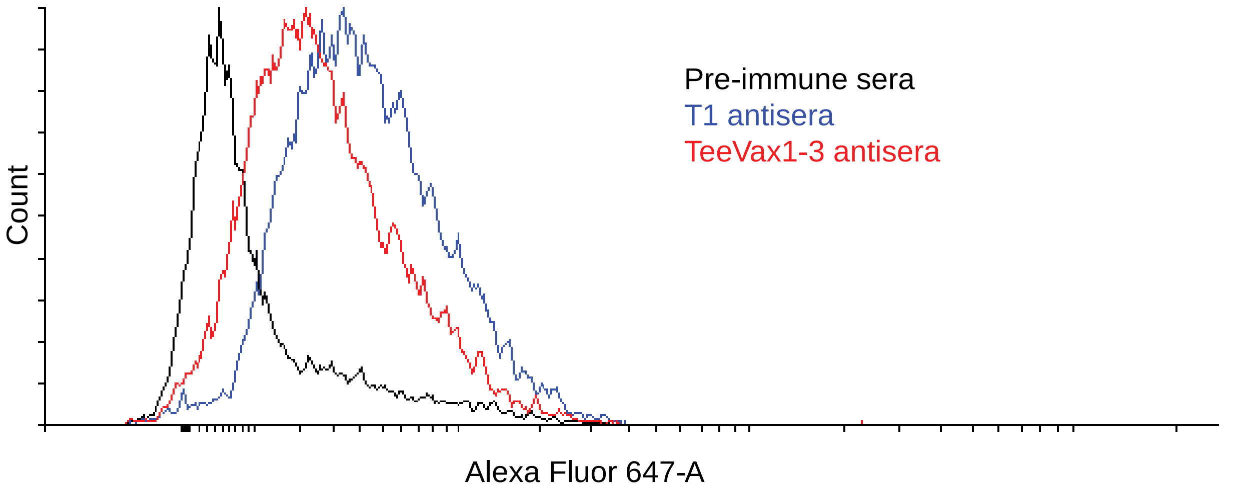

Fig. S3. Flow cytomerty showing antibody detection of native pilus on GAS.
GAS SF370 (M1T1) grown to early exponential phase was incubated with with rabbit sera and antibody-binding detected with anti-rabbit IgG- Alexa Fluor 674.

Strain	*emm*-type	*tee*-type	Origin
SF370	1	1	ATCC
5448	1	1	Clinical isolate [50]
MGAS10270	2	2	ATCC
GAS13123	78	3.1	ESR
GAS1142	65	3.2	ESR
MGAS10750	4	4	ATCC
9893	82	5	ESR
MGAS10394	6	6	ATCC
GAS10143	114	7	ESR
GAS13101	85	8	ESR
GAS13345	74	9	ESR
GAS10233	81	10	ESR
89/54	89	11	ESR
94/354	12	12	ESR
10028	41	13	ESR
GAS09426	101	14	ESR
MGAS8232	18	18.1	ATCC
57G	49	18.2	ESR
GAS11550	75	25	ESR
MGAS6180	28	28.1	ATCC
GAS11209	91	28.2	ESR

Supplementary Table 1. GAS strains used in this study.
Abbreviations: ATCC, American Type Culture Collection; ESR, Institute of Environmental Science and Research New Zealand.

Supplementary Table 2. Primers used in this study

PCR primer pair	Template	Description of product amplified
Spy0128.fw<br>cgggatccgctacaacagttcacgg	gDNA from SF370	T1-antigen
Spy0128.rv<br>cggaattcttattcaaagactttttatttg		
Spy0128.fw<br>cgggatccgctacaacagttcacgg	gDNA from MGAS10270	N-term domain of T1-antigen included in TeeVax1
M1_FctAN.rev<br>gcgaattcaagatct agtagaatctaagctatttttg		
spyM2_0109.fw<br>ggatcc gaggacaccagagtgccttctc	gDNA from MGAS10270	T2-antigen
spyM2_0109.rev<br>aagctt tta accaccagtattagggattgaagg		
LT2M.fw<br>cgggatccggcagcggtagcggcaaaaatacaacagttaatgaacctaaag	gDNA from MGAS10270	Linker + Middle domain of T2-antigen included in TeeVax3
T2M.rv<br>gcgaattcaagatcttgtatgcacttcaggaacttc		
T3.2m65.fw<br>cgggatcc gagacggcaggagtgtc	gDNA from GAS13123	T3.1-antigen
T5 M82R<br>gcgaattcaagatct agtgtcacgcttatttgtgacaac		
T3.2m65.fw<br>cgggatcc gagacggcaggagtgtc	gDNA from GAS1142	T3.2-antigen
m41m65_fctac_no sm.rev<br>gcgaattcaagatct agtgtcacgcttatttgtg		
M65_FctAC.fw<br>cgggatcc tcgttaaaagttaagaagaatg	gDNA from GAS1142	C-term domain of T3.2-antigen included in TeeVax2
m41m65_fctac_no sm.rev<br>gcgaattcaagatct agtgtcacgcttatttgtg		
spyM4_0116.fw<br>gcggatcc gaatcatcacataaaaccgatg	gDNA from MGAS10750	T4-antigen
spyM4_0116.rev<br>cggaattc tta accaccagtttgaggaatgg		
LT4N.fw<br>cgggatccggcagcggtagcggcgaatcatcacataaaaccgatg	gDNA from MGAS10750	Linker + N-term domain of T4-antigen included in TeeVax3
LT4N.rev<br>gcgtcgacttaagatctcttcgttgtggtattcttagg		
T5 M82F<br>gcggatcc gagacggcaggagttgtaac	gDNA from 9893	T5-antigen
T5 M82R<br>gcgaattcaagatct agtgtcacgcttatttgtgacaac		
M82_FctAC.fw<br>cgggatcc tccttaaaaattgaaaaacaaataac	gDNA from 9893	C-term domain of T5-antigen included in TeeVax1
M82_FctAC.rev<br>gcgaattcaagatct agttggaacttgagtgtcac		
spyM6_0160.fw<br>ggatcc ttatcaaaagatgatactgcacaac	gDNA from MGAS10394	T6-antigen
spyM6_0160.rev<br>aagctt tta tcc acctgtcgaaggtaattcacc		
T6M.fw<br>cgggatccccatctattacaaagaaagtaacc	gDNA from MGAS10394	Middle domain of T6-antigen included in TeeVax3
T6M.rev<br>gcgaattcaagatctataagtataaacaattttagaatcttctttg		
T7m114.fw<br>cgggatcc gagacggcaggggtgattg	gDNA from GAS10143	T7-antigen
M41M65_FctAC_no SM.rev<br>gcgaattcaagatct agtgtcacgcttatttgtg		
T7m114C.fw<br>gcggatcctcattagaagttaaaaag	gDNA from GAS10143	C-term domain of T7-antigen included in TeeVax2
M41M65_FctAC_no SM.rev<br>gcgaattcaagatct agtgtcacgcttatttgtg		
M85_FctAN.fw<br>cgggatcc gagacggcaggggtgac	gDNA from GAS13101	T8-antigen
M41M65_FctAC_no SM.rev<br>gcgaattcaagatct agtgtcacgcttatttgtg		
M85_FctAN.fw<br>cgggatcc gagacggcaggggtgac	gDNA from GAS13101	N-term domain of T8-antigen included in TeeVax2
M85_FctAN.rev<br>gcgaattcaagatct tgttgtatcaaaagaattttcaaata		
M74_FctAN.fw<br>cgggatcc gaaggtggagtgagtactgg	gDNA from GAS13345	T9-antigen
M41M65_FctAC_no SM.rev<br>gcgaattcaagatct agtgtcacgcttatttgtg		
M74_FctAN.fw<br>cgggatcc gaaggtggagtgagtactgg	gDNA from GAS13345	N-term domain of T9-antigen included in TeeVax2
M74_FctAN.rev<br>gcgaattcaagatct tgttgttgtaaattcgttgtc		
T10m81.fw<br>cgggatcc gagacggcaggggttgt	gDNA from GAS10233	T10-antigen
M81_FctAC.rev<br>gcgaattcaagatct agttgggataggcaagtttctc		
M81_FctAC.fw	gDNA from GAS10233	C-term domain of T10-antigen included in TeeVax2

cgggatcc tctttaactatcaaaaaagaagttac<br>M81_FctAC.rev<br>gcgaattcaagatct agttgggataggcaagtttctc		
T11 M89F<br>gcggatcc gaagtaaattatgtaaaatcaggag<br>T11 M89R<br>gcgaattcaagatct agtgtcacgcttatttgtgactg	gDNA from 89/54	T11-antigen
M89_FctAC.fw<br>gcggatcc tctttagttgttaaaaaagagg<br>M89_FctAC.rev<br>gcgaattcaagatct agttggaacttgagtgtcac	gDNA from 89/54	C-term domain of T11-antigen included in TeeVax1
T12 M12F<br>gcggatcc gagacggcaggggttgttag<br>T12 M12R<br>gcgaattcaagatct agtgtcacggttatttgtgacaac	gDNA from 94/354	T12-antigen
M12_FctAC.fw<br>gcggatcc tcattaaaaattgaaaaggaag<br>M12_FctAC.rev<br>gcgaattcaagatct agttggaacttgagtgtcac	gDNA from 94/354	C-term domain of T12-antigen included in TeeVax1
M101_FctAN.fw<br>cgggatcc gagacggcaggggttgtaacag<br>T14m101_noSM.rev<br>gcgaattcaagatct agtgtcacggttatttataactaag	gDNA from 10028	T13-antigen, also included in TeeVax2
M101_FctAN.fw<br>cgggatcc gagacggcaggggttgtaacag<br>T14m101_noSM.rev<br>gcgaattcaagatct agtgtcacggttatttataactaag	gDNA from GAS09426	T14-antigen
M101_FctAN.fw<br>cgggatcc gagacggcaggggttgtaacag<br>M101_FctAN.rev<br>gcgaattcaagatct agttgtatcaaaagaatttttaaaa	gDNA from GAS09426	N-term domain of T14-antigen included in TeeVax2
M18_0128.fw<br>cgggatccgagacagcaggagtgattg<br>M18_0128.rev<br>cggaattcttattcgtcagtagatttg	gDNA from MGAS8232	T18.1-antigen
M18_0128.fw<br>cgggatccgagacagcaggagtgattg<br>M18_FctAN.rev<br>gcgaattcaagatct agttgtatcaaaaaaatttttaaac	gDNA from MGAS8232	N-term domain of T18-antigen included in TeeVax1
spyM49_fctA.fw<br>ctagggatccgagacagcaggagtgattg<br>spyM28_0109.rev<br>cggaattcttaaacaccagttggaacttgagtg	gDNA from 57G	T18.2-antigen
T23m23F<br>gcggatccttatcaaaagatgataaggcggag<br>T23m23R<br>gcgaattcaagatctttcacctaatttggtgttaggaatttc	Synthetic DNA from IDT	T23-antigen
LT23M.fw<br>cgggatccggcagcggtagcggcccaacaattagcaagtcaattac<br>LT23M.rv<br>gcgaattcaagatctataggtgtagacggtttttttatcc	Synthetic DNA from IDT	Linker + Middle domain of T23-antigen included in TeeVax3
TEE25FWD<br>aaaggatccaataccaagtagtacggt aaggg<br>TEE25REV<br>aaagaattcttattcaggacgtttgtt gttttctac	gDNA from GAS11550	T25-antigen
LT25M.fw<br>cgggatccggcagcggtagcggcccaaaaccgggtaaggatg<br>T25M.rv<br>gcgaattcaagatcttgtatgaacttcaggattatctgaag	gDNA from GAS11550	Linker + Middle domain of T25-antigen included in TeeVax3
spyM28_0109.fw<br>gcggatcc gagacggcaggagtgtccgaaaatg<br>spyM28_0109.rev<br>cggaattcttaaacaccagttggaacttgagtg	gDNA from MGAS6180	T28.1-antigen
spyM28_0109.fw<br>gcggatcc gagacggcaggagtgtccgaaaatg<br>M28_FctAN.rev<br>gcgaattcaagatct agttgtcttaaaggagttttc	gDNA from MGAS6180	N-term domain of T28.1-antigen included in TeeVax1
TEE28.1FWD<br>aaaggatcctgagacggcaggggtaac taa<br>TEE28.1REV<br>aaagaattcttattcacggttatttgt gacaacgat	gDNA from GAS11209	T28.2-antigen
